# Enhanced ROS Production and Mitochondrial Metabolic Shifts in CD4^+^ T Cells of an Autoimmune Uveitis Model

**DOI:** 10.3390/ijms252111513

**Published:** 2024-10-26

**Authors:** Ronja Söth, Anne L. C. Hoffmann, Cornelia A. Deeg

**Affiliations:** Chair of Physiology, Department of Veterinary Sciences, Ludwig Maximilian University of Munich, D-82152 Martinsried, Germany

**Keywords:** equine recurrent uveitis (ERU), autoimmune uveitis, neuroinflammation, autoimmunity, immunometabolism, CD4^+^ T cell, reactive oxygen species, metabolic flexibility

## Abstract

Equine recurrent uveitis (ERU) is a spontaneously occurring autoimmune disease and one of the leading causes of blindness in horses worldwide. Its similarities to autoimmune-mediated uveitis in humans make it a unique spontaneous animal model for this disease. Although many aspects of ERU pathogenesis have been elucidated, it remains not fully understood and requires further research. CD4^+^ T cells have been a particular focus of research. In a previous study, we showed metabolic alterations in CD4^+^ T cells from ERU cases, including an increased basal oxygen consumption rate (OCR) and elevated compensatory glycolysis. To further investigate the underlying reasons for and consequences of these metabolic changes, we quantified reactive oxygen species (ROS) production in CD4^+^ T cells from ERU cases and compared it to healthy controls, revealing significantly higher ROS production in ERU-affected horses. Additionally, we aimed to define mitochondrial fuel oxidation of glucose, glutamine, and long-chain fatty acids (LCFAs) and identified significant differences between CD4^+^ T cells from ERU cases and controls. CD4^+^ T cells from ERU cases showed a lower dependency on mitochondrial glucose oxidation and greater metabolic flexibility for the mitochondrial oxidation of glucose and LCFAs, indicating an enhanced ability to switch to alternative fuels when necessary.

## 1. Introduction

The metabolism of immune cells in autoimmune diseases has attracted increasing interest in recent years. Depending on metabolic needs and activation status, immune cells can quickly adapt and switch between dominating metabolic pathways [1,2]. These include the two primary pathways for generating adenosine triphosphate (ATP): oxygen-consuming oxidative phosphorylation (OXPHOS) in the mitochondria and glycolysis in the cytoplasm, where glucose is converted to pyruvate [1,3]. This pyruvate can then either enter the tricarboxylic acid (TCA) cycle in the mitochondria or undergo conversion to lactate in the cytoplasm, which is independent of oxygen [1,3]. Metabolism of glucose to lactate was originally thought to occur only under anaerobic conditions until Otto Warburg discovered the Warburg effect, demonstrating that cancer cells switch from OXHPHOS to glycolysis for ATP production even in the presence of sufficient oxygen [4]. This process, known as ‘aerobic glycolysis’, is also observed in activated immune cells [1,5,6]. This metabolic reprogramming enables immune cells to achieve full effector function by rapidly generating energy to meet the increased demands for proliferation, while also providing pyruvate as a key metabolic intermediate [7,8]. For example, in both humans and mice, activated T helper 1 (Th1), Th2, and Th17 cells primarily rely on glycolysis [9], whereas naïve T cells and anti-inflammatory cells such as memory CD8^+^ T cells and T regulatory cells (Tregs) prefer OXPHOS and fatty acid oxidation (FAO) for ATP production [2,10,11]. Glucose, glutamine, and fatty acids are the primary energy substrates for immune cells, including CD4^+^ T cells [6]. End products of their metabolism feed into the TCA cycle, where they are oxidized to CO_2_, generating electrons that fuel the electron transport chain (ETC) via NADH and FADH2 [1]. Electron movement through ETC complexes I, III, and IV drives proton (H^+^) transfer across the inner mitochondrial membrane, creating a proton gradient that delivers the energy needed for ATP production by complex V [12]. The entirety of this process is referred to as OXPHOS, which is elevated in activated CD4^+^ T cells, but with the OXPHOS/glycolysis ratio being lower in activated compared to naïve murine CD4^+^ T cells [1,13].

Elevated OXPHOS in immune cells not only provides increased energy, but also contributes to a greater generation of reactive oxygen species (ROS), primarily produced by complexes I and III of the ETC [11,14]; other significant sources of mitochondrial ROS (mROS) include enzymes such as the alpha-ketoglutarate dehydrogenase and pyruvate dehydrogenase complexes [15,16,17]. Non-mitochondrial sources of ROS that should be considered include the NADPH oxidase (NOX) family, which is a major producer of cytoplasmic ROS, as well as the endoplasmic reticulum and peroxisomes [15,18,19]. These ROS act as key signaling molecules in T cell activation [11,15,20]. However, excessive ROS production can cause oxidative stress, leading to mitochondrial damage, dysfunction, and altered metabolism [21]. These double-sided effects of ROS are thought to contribute to the development of autoimmune diseases [15,22]. For example, in a recent study using a murine model for psoriasis, Nicotinamide mononucleotide (NMN), a precursor of nicotinamide adenine dinucleotide (NAD^+^), significantly reduced ROS levels, mitochondrial dysfunction, and autoimmune inflammation in HEKa and HaCaT cells [23]. Novel strategies to target ROS production and the oxidative stress they induce are continuously emerging.

Autoimmune uveitis in humans is a sight-threatening disease characterized by recurring episodes of intraocular inflammation [24,25]. Pathogenesis involves the breakdown of the blood-retinal barrier, allowing autoreactive cells, predominantly CD4^+^ T cells, to infiltrate the immune-privileged eye and cause neuroinflammation [26,27,28]. Equine recurrent uveitis (ERU) serves as a spontaneous animal model for studying autoimmune uveitis due to its strong similarities in disease onset and pathogenesis [29,30]. Like human autoimmune uveitis, ERU exhibits a spontaneous onset and a remitting-relapsing nature, with the involvement of autoreactive lymphocytes [31,32]. These parallels, along with the accessibility of peripheral blood cells from ERU-affected horses that are not undergoing systemic treatment, establish ERU as a valuable translational model for autoimmune uveitis [30,33,34]. Thus, studying peripheral blood cells from ERU cases is beneficial for exploring the molecular mechanisms underlying the development of both ERU and autoimmune uveitis.

In previous studies performed in our research group, peripheral blood mononuclear cells (PBMCs) and CD4^+^ T cells from ERU cases showed a distinct metabolic phenotype compared to healthy control cells, with one of these alterations being an increased OXPHOS [35]. In addition, our work group discovered that CD4^+^ T cells from ERU cases exhibited decreased basal glycolysis [35]. However, after mitochondrial inhibition, glycolysis was increased to compensate for the energy deficit, with CD4^+^ T cells from ERU cases showing significantly higher rates of compensatory glycolysis [35]. Since CD4^+^ T cells are the driving force of ERU and exhibit the metabolic changes described, we decided to continue focusing on this cell population.

Our study aimed to unravel potential differences in ROS production and mitochondrial metabolism, particularly focusing on the oxidation of glucose, glutamine, and fatty acids. Variations in the metabolism of auto-aggressive cells may help to identify future therapeutic targets for autoimmune diseases such as ERU and autoimmune uveitis at a molecular level, while also contributing to understanding the complex relationship between metabolism and neuroinflammation.

## 2. Results

### 2.1. CD4^+^ T Cells from ERU Cases Showed Increased Production of ROS After Stimulation with PWM

**Figure 1:**
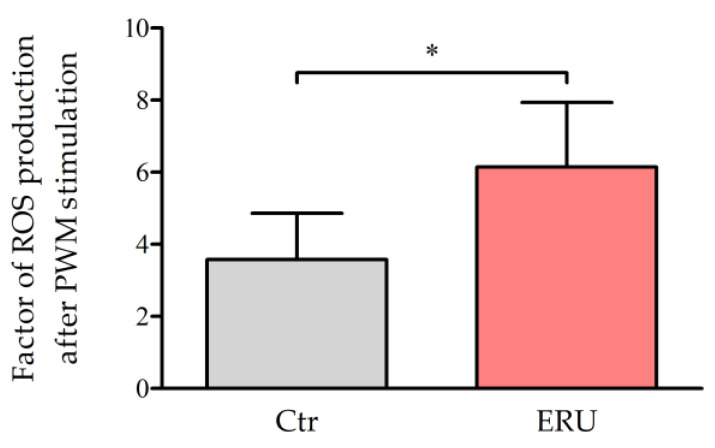
CD4^+^ T cells from ERU cases produced higher levels of reactive oxygen species (ROS) after stimulation with pokeweed mitogen (PWM). Bar graphs display the factor of ROS production in CD4^+^ T cells from healthy horses (Ctr, gray, *n* = 6) and equine recurrent uveitis cases (ERU, red, *n* = 5) after 90 min of stimulation with PWM. The mean value is shown as a factor and the error bars correspond to the standard deviation. The factor of ROS production in control cells was determined at 3.6, while ERU cases exhibited a factor of 6.1. To compare the factor of ROS production between CD4^+^ T cells from ERU cases and healthy control cells, Student’s *t*-test was used (* *p* = 0.023).

To determine whether the elevated OXPHOS in CD4^+^ T cells from ERU cases [35] leads to higher ROS production, we measured ROS production after stimulation of CD4^+^ T cells from healthy controls and ERU horses with the polyclonal T and B cell mitogen Pokeweed Mitogen (PWM) [36]. Using flow cytometry, ROS production was visualized with 2′,7′-Dichlorofluorescein diacetate (DCF-DA) staining, which converts to fluorescent 2′,7′-Dichlorofluorescein upon oxidation by ROS. In controls, the mean factor of ROS production after PWM stimulation was calculated at 3.6, while cells from ERU cases exhibited a significantly higher ROS production with a mean factor of 6.1, representing a 1.7-fold increase in ROS production in ERU cases (Figure 1). In a mouse model with reduced mitochondrial reactive oxygen species (mROS) production in T cells, it has been demonstrated that ROS from mitochondrial complex III serve as signaling molecules essential for T cell activation [14,37]. However, in excessive amounts, ROS have been observed to cause mitochondrial dysfunction [38]. For example, this has been demonstrated in CD4^+^ T cells of humans living with HIV who are undergoing antiretroviral therapy [38]. Therefore, we decided to look more closely at the mitochondrial metabolism of CD4^+^ T cells from ERU cases in the following experiments.

### 2.2. Reduced Dependency on and Higher Flexibility for Mitochondrial Glucose Oxidation in CD4^+^ T Cells from ERU Cases

**Figure 2:**
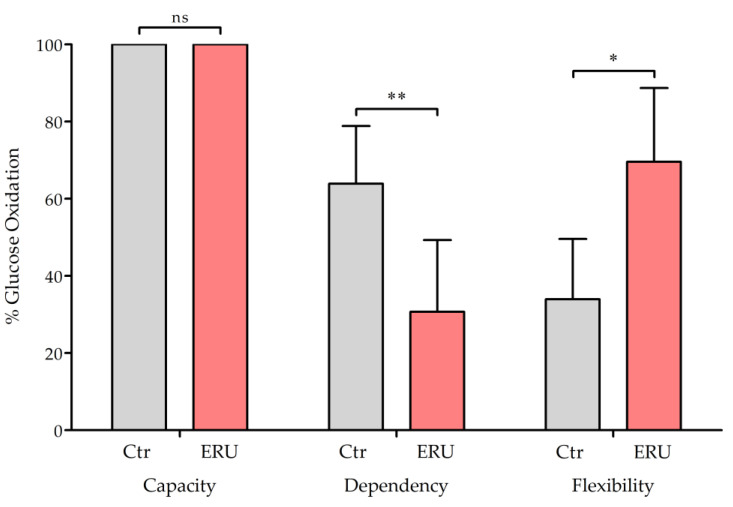
Lower dependency on and higher flexibility for mitochondrial oxidation of glucose in ERU cases. Bar graphs show the percentage of dependency on mitochondrial glucose oxidation in CD4^+^ T cells from healthy controls (gray, *n* = 6) and ERU cases (red, *n* = 5) as well as capacity and flexibility from healthy controls (gray, *n* = 5) and ERU cases (red, *n* = 5). The mean value is presented as a percentage, and the error bars correspond to the standard deviation. Both groups showed 100% capacity to use glucose for mitochondrial oxidation (ns = not significant), whereas CD4^+^ T cells from ERU cases were significantly (** *p* = 0.009) less dependent and exhibited significantly (* *p* = 0.012) greater flexibility to use glucose as a mitochondrial fuel compared to controls. Student’s *t*-test was used to test for statistical significance.

To investigate the underlying reasons for and consequences of the previously observed differences in CD4^+^ T cell metabolism in ERU, such as the increased basal oxygen consumption rate (OCR), lower basal glycolytic rates, increased compensatory glycolysis [35], and greater production of ROS (Figure 1), we decided to define mitochondrial fuel utilization of glucose in CD4^+^ T cells from ERU cases. Both healthy horses and ERU cases showed 100% capacity to use glucose as a mitochondrial fuel, which describes the ability of the cell’s mitochondria to oxidize a fuel when other fuel pathways are inhibited (Figure 2). The dependency on mitochondrial glucose oxidation indicates the extent to which specific cells rely on this fuel source for energy production and their inability to compensate by oxidizing alternative fuels. The dependency on glucose oxidation was significantly lower in CD4+ T cells from ERU cases (ERU = 30.7%) compared to controls (Ctr = 66.05%), suggesting that mitochondria in CD4+ T cells from ERU cases have an enhanced ability to compensate for impaired glucose oxidation by utilizing alternative fuel sources. Since flexibility is calculated by subtracting dependency from capacity, this resulted in greater flexibility in ERU cases (ERU = 69.3%) to use glucose for mitochondrial respiration compared to controls (Ctr = 34.0%) (Figure 2). We hypothesized that a lower dependency on glucose oxidation requires these cells to increase their ability to use alternative mitochondrial fuels such as amino acids or fatty acids. Therefore, we subsequently focused on validating this potential metabolic shift.

### 2.3. Glutamine Metabolism Showed No Significant Differences Between ERU Cases and Controls

**Figure 3:**
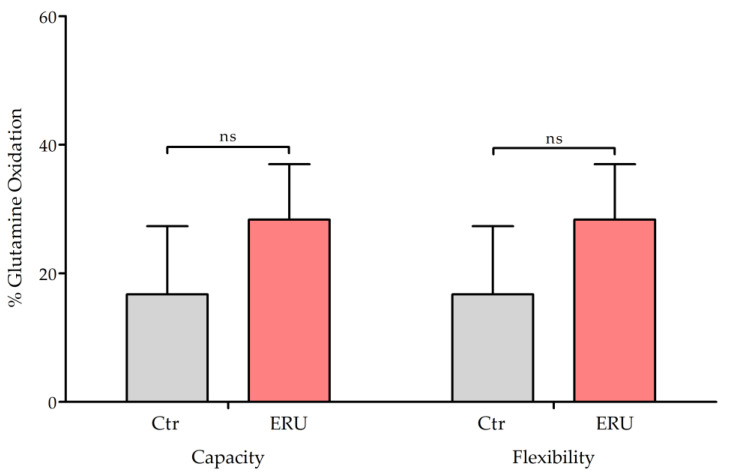
Mitochondrial fuel utilization of glutamine did not show significant differences in CD4^+^ T cells from ERU cases. Bar graphs show the percentage of capacity and flexibility of mitochondrial glutamine oxidation in CD4^+^ T cells from healthy controls (gray, *n* = 6) and ERU cases (red, *n* = 5). Flexibility was calculated by subtracting the dependency from the capacity (flexibility% = capacity% − dependency%). The mean value is shown as a percentage and the error bars correspond to the standard deviation. Student’s *t*-test revealed no statistically significant (ns *p* = 0.066) differences in capacity or flexibility of substrate utilization of glutamine.

To determine whether CD4^+^ T cells from ERU cases compensated for their observed lower dependency on mitochondrial glucose metabolism (Figure 1) by upregulating amino acid oxidation, we determined the dependency, capacity, and flexibility of these cells for glutamine oxidation. Mitochondrial respiration was measured following inhibition of the glutamine oxidation pathway, as well as after blocking glucose and fatty acid oxidation pathways, to evaluate how these cells adapt their metabolic processes. Differences in capacity (ERU = 31.3%, Ctr = 16.7%) and flexibility (ERU = 31.3%, Ctr = 16.7%) between ERU cases and controls were not significant. Data on dependency were measured at 0% in all animals, resulting in flexibility directly corresponding to capacity (Figure 3).

### 2.4. CD4^+^ T Cells from ERU Cases Showed Increased Capacity and Flexibility for Mitochondrial Oxidation of Long-Chain Fatty Acids

**Figure 4:**
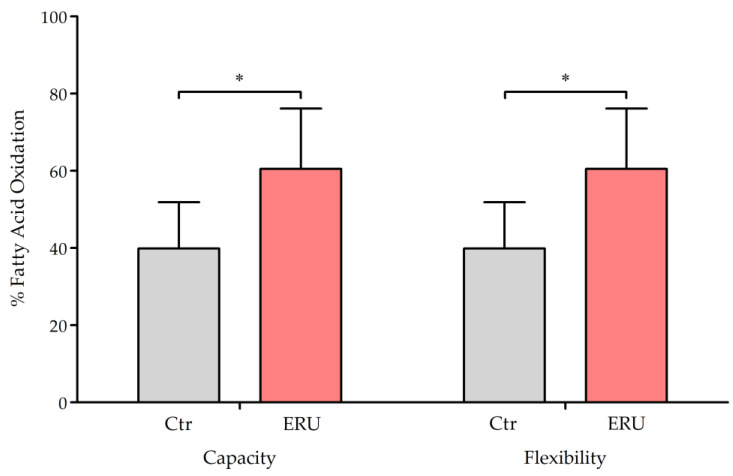
CD4^+^ T cells from ERU cases showed greater capacity and flexibility to utilize long-chain fatty acids (LCFAs) for mitochondrial energy production. Bar graphs represent the percentage of capacity and flexibility for mitochondrial oxidation of LCFAs in CD4^+^ T cells from healthy controls (gray, *n* = 6) and ERU cases (red, *n* = 5). Flexibility was calculated by subtracting the dependency from the capacity (flexibility% = capacity% − dependency%). The mean value is shown as a percentage and the error bars correspond to the standard deviation. ERU cases showed significantly (* *p* = 0.041) higher capacity and flexibility to use LCFAs for mitochondrial oxidation. Student’s t-test was used to test for statistical significance.

Our next step was to investigate whether fatty acids were used to fuel OXPHOS at a higher rate in CD4^+^ T cells from ERU cases to compensate for the lower dependency on mitochondrial glucose oxidation (Figure 1). Long-chain fatty acid (LCFA) metabolism was inhibited by blocking carnitine palmitoyl-transferase 1a (CPT1a) of the outer mitochondrial membrane using Etomoxir. This enzyme is crucial for transporting palmitate, an LCFA, into the mitochondrion [39]. The decrease in OCR was measured after inhibiting both the fatty acid oxidation pathway and the alternative pathways of glucose and glutamine oxidation. The analysis revealed no dependency on mitochondrial LCFA oxidation in CD4^+^ T cells from ERU cases (ERU = 0%) and controls (Ctr = 0%). Consequently, these cells were able to fully compensate for the blocked utilization of LCFAs. The capacity to use LCFAs for mitochondrial respiration was significantly higher in ERU cases (ERU = 62.8%) compared to controls (Ctr = 39.9%). Since the dependency on fatty acids was 0% in all animals, flexibility also corresponded to capacity (ERU = 62.8%, Ctr = 39.9%) (Figure 4), revealing an increased ability of CD4^+^ T cells from ERU cases to use LCFAs for mitochondrial respiration.

## 3. Discussion

The metabolism of CD4^+^ T cells has been extensively studied to elucidate the pathogenesis of various autoimmune diseases, including autoimmune-mediated uveitis in humans [6,25]. Autoimmune uveitis is characterized by non-infectious ocular inflammations with recurring and remitting episodes, driven by CD4^+^ T cells crossing the blood-retinal barrier and contributing to inflammation [25]. Despite species differences, there are notable parallels in the pathogenesis of ERU and autoimmune uveitis, along with significant similarities in immune system composition and function [40], making the horse a valuable model for studying autoimmune-mediated uveitis [29,30,33]. Although many aspects of CD4^+^ T cell metabolism in ERU-affected horses still remain unanswered, some metabolic alterations have already been identified. Based on our previous findings of increased OXPHOS activity in CD4^+^ T cells from ERU cases [35], we aimed to determine whether this elevated OXPHOS led to enhanced ROS production in these cells. We demonstrated that CD4^+^ T cells from ERU cases produced significantly higher amounts of ROS compared to control cells following stimulation with PWM (Figure 1). Given that an activated metabolism with heightened oxygen consumption has been shown to be accompanied by elevated ROS production via the ETC complexes in both humans and mice [11,14,41], these new findings in equine CD4^+^ T cells are consistent with our previous study [35]. However, it is important to acknowledge that the DCF-DA probe used in this study is not exclusively specific to mitochondria-derived ROS [42]. Upon T cell activation, the ETC complexes generate ROS in the mitochondria, while NOX proteins simultaneously produce substantial amounts of cytoplasmic ROS, supporting ROS signaling during activation [11,15,43,44]. Consequently, other sources, such as cytoplasmic NOX proteins and peroxisomes, also contribute to overall ROS production [11,18]. Nevertheless, studies have demonstrated that DCF-DA can effectively detect mitochondria-derived ROS [45,46]. Given our previous finding of elevated OXPHOS [35], we hypothesize that the majority of the detected ROS are of mitochondrial origin. To determine which specific cellular compartments contributed to the elevated ROS levels, future studies will be necessary. For example, MitoSOX Red is a dye used to measure superoxide production in the mitochondrial matrix, though its use has limitations, including cyto- and mitotoxicity [42]. Subsequent studies could include a differential proteome analysis of isolated CD4^+^ T cell mitochondrial proteins, a highly sensitive and targeted approach with the potential to provide detailed insights into altered proteins associated with ROS in CD4^+^ T cells from ERU-affected horses.

ROS play a crucial role in various cellular functions [14,47]. Upon T cell receptor (TCR) activation, ROS signaling helps regulate T cell activation through several mechanisms [11]. These include the regulation of transcription factors and the modulation of signaling pathways such as the nuclear factor-kappa B (NF-κB) pathway, which was demonstrated in human T cells [11,48]. For example, mROS generated by complex III of the ETC are essential for T cell activation through nuclear factor of activated T cells (NFAT) [37]. This was proved by Sena et al. in research involving *Uqcrfs-*knock-out mice with a T cell-specific reduction in Rieske iron sulfur protein (RISP), a subunit of mitochondrial complex III, which led to reduced mROS production in T cells and impaired T cell activation through NFAT [37]. These mice exhibited an inability to induce antigen-specific T cell expansion in vivo, highlighting the crucial role of mROS in T cell activation [37]. Notably, another study in mice with superoxide-deficient T cells showed a reduced Th1 cell response [49]. In line with this, the elevated ROS production observed in CD4^+^ T cells from ERU cases may contribute to their activated pro-inflammatory Th1 phenotype, as indicated by the increased expression of interferon-gamma (IFNγ) in CD4^+^ T cells from ERU horses [50].

A link between elevated ROS levels and the Th17/Treg balance has been explored in CD4^+^ T cells from patients with *Chlamydia psittaci pneumonia* (CPP). Compared to healthy controls, CPP patients exhibited higher ROS levels in peripheral blood, an increased abundance of Th17 cells, and a reduced abundance of Tregs [51]. Treatment with H_2_O_2_ led to an increase in Th17 cell differentiation and a further decrease in Treg differentiation, indicating that ROS play a role in regulating the balance between Th17 and Treg cells in CPP [51]. This relationship has also been observed in other models. For instance, in a murine model of asthma, elevated ROS levels and a disrupted Th17/Treg balance were identified, while treatment with vitamin D downregulated ROS levels and restored this balance [52]. Additionally, in Henoch-Schonlein purpura, a form of recurrent immunoglobulin A-mediated vasculitis, increased ROS levels have similarly been linked to a Th17/Treg imbalance [53]. Th17 cells are considered key players in the development of various autoimmune diseases, including rheumatoid arthritis (RA) and systemic lupus erythematosus (SLE) [54]. This is supported by the presence of interleukin-17 (IL-17), the hallmark cytokine of Th17 cells, in the synovial fluid of RA patients, which has been associated with disease severity [54,55]. Moreover, SLE patients exhibit increased IL-17 production compared to healthy controls, with plasma IL-17 levels showing a positive correlation with disease activity [54,56]. These findings underscore the crucial role of Th17 cells in autoimmune diseases [54]. We hypothesize that the elevated ROS production observed in CD4^+^ T cells from ERU-affected horses may similarly influence the Th17/Treg balance. This hypothesis warrants further investigation. While Th17 cells have not been directly identified in horses, their involvement in ERU is suggested by matching cytokine patterns in equine iris and ciliary bodies [57], which align with findings from rodent models for experimental autoimmune uveitis (EAU), where Th17 cells have been implicated in driving inflammatory processes in autoimmune uveitis [58]. In particular, Th17/Treg balance, with Th17 cells promoting inflammation and Tregs working to control it, is being actively investigated in the context of autoimmune disease development [10,15,59]. During acute inflammatory episodes, Tregs were found to be less abundant in the peripheral blood of patients with autoimmune uveitis compared to healthy controls and patients in clinical remission [59,60]. Consequently, Tregs are believed to play a role in the remitting-relapsing nature of autoimmune uveitis [59,60,61]. To our knowledge, these differences have not yet been detected in the peripheral blood of ERU-affected horses. However, the involvement of Tregs in ERU disease remission remains very likely [61], making the effect of ROS on Th17/Treg balance a compelling area of research for both ERU and autoimmune uveitis. Moreover, the potential impact of the interleukin-33/interleukin-31 (IL-33/IL-31) axis should also be considered in this context. A study in lupus-prone mice demonstrated that IL-33 inhibition can reduce SLE progression by promoting Treg expansion and suppressing Th17 cells [62,63]. In patients with Behçet’s disease-associated uveitis, elevated serum levels of IL-33 and IL-31 have been reported, with IL-33 playing a role in the progression of experimental autoimmune uveitis (EAU) [62,64]. However, the precise role of the IL-33/IL-31 axis in equine recurrent uveitis (ERU) and autoimmune uveitis remains unclear and warrants further investigation.

Oxidative stress, which results from an imbalance between ROS and the ability of cellular antioxidant mechanisms to neutralize them [65], has already been identified as a factor that exacerbates disease progression in a murine model of EAU [66,67]. ROS-deficient mice exhibited lower EAU disease scores compared to wild-type mice [68]. Additionally, these mice showed reduced cytokine levels in the retina and decreased NF-κB activity, suggesting that similar mechanisms may be relevant to autoimmune uveitis in humans [68]. However, the validation of higher ROS production in our spontaneous equine animal model [34] of autoimmune uveitis validates the role of oxidative stress in this disease and lays the groundwork for further investigations into the metabolic consequences of these ROS.

When ROS production becomes excessive, it can cause cellular damage, particularly to the mitochondria, leading to mitochondrial dysfunction [21,38]. The inability of mitochondria to function properly causes impaired energy production, disruption of OXPHOS, increased production of mROS, and an enhanced inflammatory response [15,69,70]. A similar effect was observed in CD4^+^ T cells from patients with SLE [41]. It was hypothesized that this resulted from mitochondrial damage caused by the chronic activation of these cells, leading to a less efficient metabolism and greater ROS generation [41]. This exhausted phenotype could be hypothesized for CD4^+^ T cells from ERU cases as well, providing an alternative explanation for the enhanced OXPHOS.

To summarize, increased ROS production in CD4^+^ T cells from ERU cases may result in chronic activation, promote Th1 and Th17-mediated inflammation, disrupt the Th17/Treg balance, and induce mitochondrial damage, leading to an altered mitochondrial metabolism. Further studies are needed to determine whether the elevated ROS production in these cells has resulted in a generally more active and efficient metabolism, or if chronic activation has caused mitochondrial impairments leading to the observed changes. For example, experiments to assess OXPHOS effectiveness through proton leak measurements could provide additional insights [41].

Based on activation status, oxygen availability, or to compensate for impairments in mitochondrial ATP production, immune cells have to undergo significant alterations in their metabolism [1,71]. To identify alterations in mitochondrial metabolism potentially associated with the elevated ROS levels in CD4^+^ T cells from ERU cases, we analyzed mitochondrial utilization of glucose, glutamine, and fatty acids of respective cells, as these are the primary metabolic fuel sources in immune cells [6]. The capacity to use glucose for mitochondrial oxidation was 100% in both ERU cases and controls, indicating that the cells’ mitochondria retained their full ability to oxidize glucose when other fuel pathways were inhibited (Figure 2). Interestingly, CD4^+^ T cells from ERU cases exhibited a significantly lower dependency on mitochondrial glucose oxidation compared to control cells (Figure 2). We hypothesize that this is due to the elevated levels of aerobic glycolysis previously observed in CD4^+^ T cells from ERU cases [35]. These cells appear to favor the breakdown of glucose to lactate in the cytoplasm rather than oxidizing glucose to CO₂ via pyruvate in the mitochondria. Mitochondrial oxidation of pyruvate is more efficient, providing higher rates of ATP through OXPHOS [72]. However, glycolysis is faster and supplies pyruvate as an important metabolic intermediate, making it the preferred pathway for activated, proliferating cells [7]. These alterations resulted in a greater flexibility for glucose oxidation in CD4^+^ T cells from ERU cases, supporting the hypothesis of an activated and metabolically flexible phenotype of these cells [35] (Figure 2).

Although we demonstrated that CD4^+^ T cells from ERU cases did not show a significantly different glutamine oxidation compared to controls (Figure 3), the capacity to use glutamine as a mitochondrial fuel was still slightly higher in cells from ERU cases (ERU = 31.25%, Ctr = 16.77%, ns *p* = 0.0662), suggesting that these cells may nevertheless utilize amino acids for mitochondrial oxidation to a greater extent, which merits further investigations. This observation aligns with the known behavior of activated T cells, for instance Th1 cells, which depend on glutaminolysis to support proliferation and IFNγ production [1,73]. Specifically, activated T cells have been shown to increase the expression of glutamine transporters [74] and glutamine starvation has been demonstrated to reduce T cell proliferation and cytokine secretion [1,75]. Our data show 0% dependency on glutamine oxidation in both ERU cases and controls, which means that these cells were able to fully compensate for the blocked glutamine pathway by oxidizing alternative fuels, such as glucose and LCFAs.

Regarding these alternative fuels, we were able to demonstrate that CD4^+^ T cells from ERU cases showed a significantly higher capacity and flexibility for the mitochondrial oxidation of LCFAs (Figure 4). The dependency on mitochondrial FAO was calculated at 0%, indicating that these cells could fully compensate for blocked FAO by using alternative fuels for mitochondrial respiration. The 0% dependency on glutamine oxidation and FAO is consistent with the fact that these cells had a 100% capacity to utilize glucose for mitochondrial oxidation when other pathways were inhibited. Therefore, any dependency on other fuel sources would be contradictory. A limitation of these findings is that they do not extend to short- and medium-chain fatty acids (SCFAs, MCFAs), which can enter the mitochondria through passive diffusion [76,77]. Consequently, the inhibition of CPT1a with Etomoxir did not affect the mitochondrial oxidation of SCFAs and MCFAs. This may have contributed to the 100% capacity for glucose oxidation, since molecules that enter the mitochondrion via passive diffusion were not addressed in this study. However, as indicated by the significantly higher capacity, CD4^+^ T cells from ERU cases appeared to utilize LCFAs for mitochondrial oxidation to a greater extent. This might provide an additional explanation for the observed lower dependency on glucose oxidation (Figure 2). We conclude that the increased ability of these cells to compensate for blocked mitochondrial glucose oxidation could potentially be explained by elevated aerobic glycolysis levels [35], as well as a higher capacity and flexibility for mitochondrial FAO (Figure 4), or the utilization of different alternative fuel sources.

We theorize that the increased capacity and flexibility identified for FAO in CD4^+^ T cells from ERU cases may be attributed to the chronic activation of these cells, which attempt to meet the high energy demands associated with activation. For that purpose, cells may upregulate FAO, which produces more than twice as many ATP molecules through mitochondrial oxidation compared to glucose or amino acid catabolism [78]. This effect was observed in B-cell lymphomas, which upregulated mitochondrial FAO, likely to meet the high energy demands for rapid growth [79,80]. Furthermore, in CD4^+^ tissue-resident memory T (T_RM_) cells from Crohn’s disease (CD) patients, an elevated FAO phenotype has been detected, likely due to NF-κB pathway activation [81]. This activation increases the transcription of genes involved in FAO, contributing to a pro-inflammatory and apoptosis-resistant phenotype in these cells [81]. Considering the Th17/Treg balance, the discovery of an enhanced capacity to oxidize LCFAs in CD4^+^ T cells from ERU cases is particularly intriguing. In a murine model for autoimmune encephalomyelitis (EAE), LCFAs have been shown to promote the differentiation of naïve T cells into Th1 and Th17 cells in vivo, thereby exacerbating the disease [10,82]. Conversely, SCFAs have been found to promote Treg expansion, which improved EAE symptoms [10,82]. In summary, the elevated capacity to oxidize LCFAs in CD4^+^ T cells from ERU cases may lead to enhanced energy production, contribute to a pro-inflammatory and apoptosis-resistant phenotype, and promote the expansion of Th1 and Th17 cells in ERU.

Considering therapeutic perspectives, the observed differences in ROS production and mitochondrial oxidation of glucose and fatty acids provide multiple avenues that require further investigation and refinement. Inhibition of FAO through Etomoxir should be explored for ERU-affected horses, as this successfully reversed the FAO-driven altered phenotype in CD4^+^ T_RM_ cells in Crohn’s disease by reducing their inflammatory capacity and enhancing their apoptosis susceptibility [81]. Modulating the oxidation of LCFAs and SCFAs in CD4^+^ T cells also holds promise for the treatment of autoimmune uveitis and ERU by regulating the Th17/Treg balance, potentially enhancing the immunoregulatory effects of Tregs [10,15,59]. Furthermore, we hypothesize that inhibition of mitochondrial respiration or ROS production may offer a strategy to prevent the activation and differentiation of autoreactive T cells, like Th1 and Th17 cells, in ERU-affected horses before they become activated in the peripheral bloodstream and infiltrate the eye. Inhibition of ROS production might also be beneficial in regulating the Th17/Treg balance and preventing the activation of NF-κB [11,81,83]. For example, Fe-curcumin nanozymes, which consist of natural curcumin molecules and ferric ions with superoxide dismutase (SOD)-like enzyme properties, have been tested in a rat model of EAU [66]. In this model, Fe-curcumin effectively suppressed the inflammatory cytokine response of IFNγ, IL-17, and tumor necrosis factor-alpha (TNF-α), reduced H_2_O_2_ release, and inhibited the differentiation of Th1 and Th17 cells [66]. Additionally, vitamin D has gained recognition for its immunomodulatory effects, with low levels being associated with increased severity in Alzheimer’s disease [84]. Furthermore, vitamin D supplementation has demonstrated protective effects against oxidative stress in diabetic retinopathy [85]. These findings position vitamin D as a promising subject for further research in the treatment of autoimmune diseases linked to oxidative stress, such as ERU and autoimmune uveitis.

In summary, inhibition of mitochondrial FAO, modulation of the LCFA/SCFA ratio, and inhibition of mitochondrial respiration and ROS production represent promising options for influencing CD4^+^ T cell metabolism in ERU-affected horses. These approaches require intensive research to explore their potential for therapeutic intervention in both ERU and autoimmune uveitis.

We conclude that the observed lower dependency on mitochondrial glucose oxidation as well as the higher flexibility for mitochondrial oxidation of glucose and LCFAs might be attributed to an activated metabolic phenotype and higher metabolic flexibility of CD4^+^ T cells from ERU cases. This flexibility allows these cells to adapt and sustain energy production even when specific metabolic pathways are inhibited. A higher metabolic flexibility was previously hypothesized for CD4^+^ T cells from ERU cases, as these cells exhibited lower basal glycolytic rates, but upon inhibition of mitochondrial respiration, demonstrated increased compensatory glycolysis compared to controls [35]. This suggests that CD4^+^ T cells from ERU cases are capable of shifting their metabolic pathways to maintain energy production, highlighting a metabolically flexible phenotype. This flexibility is likely advantageous for sustaining the energy demands necessary for driving an autoimmune response, highlighting the adaptive capabilities of CD4^+^ T cells in the context of autoimmune diseases and contributing to their persistence and pathogenicity in conditions like uveitis. The elevated ROS production in CD4^+^ T cells from ERU cases is likely both a cause and a consequence of the metabolic alterations, making it a promising target for therapeutic interventions.

Further research is necessary to uncover the underlying causes of the observed metabolic changes, identify the specific cell population responsible, and develop therapeutic strategies to remodel the metabolism of CD4^+^ T cells in ERU cases, with a focus on the already identified alterations in glycolysis, OXPHOS, FAO, and ROS production.

## 4. Materials and Methods

### 4.1. Animals, Isolation of Primary Peripheral Blood Mononuclear Cells, and Ethics Approval for Animal Research

In total, blood from 10 different control horses and 10 different ERU cases was collected for the experiments within this study. Two of the control horses were used in both assays, the other horses were used in either DCF-DA staining or Seahorse XF Mito Fuel Flex Tests. More precisely, CD4^+^ T cells from 6 controls and 5 ERU cases were used for the DCF-DA staining. CD4^+^ T cells from 6 controls and 5 ERU cases were analyzed in the Seahorse XF Mito Fuel Flex Test. All ERU cases were in a quiescent stage of disease during blood withdrawal. Diagnosis of ERU was conducted by experienced clinicians at the Equine Hospital of LMU Munich, based on typical clinical signs characteristic for uveitis and a documented health history of multiple (two or more) episodes of inflammation in the affected eye that are pivotal signs of ERU [31]. At the time of blood withdrawal, no clinical symptoms of uveitis were present, as these horses were in the quiescent stage of ERU, allowing us to rule out pathogen-associated endophthalmitis. The quiescent intervals between inflammatory episodes can vary from weeks to several months, gradually shortening over time [86]. During acute uveitic episodes, horses are treated with systemic nonsteroidal anti-inflammatory drugs (NSAIDs) and topical immunosuppressive agents but remain untreated during quiescent periods [31,87,88]. Systemic immunosuppressive therapy is reserved for severe cases that are unresponsive to conventional anti-inflammatory treatment [29]. Given the relatively long intervals between acute uveitic episodes [86], the short elimination half-life of NSAIDs [89], and the topical application of corticosteroids [90], these drugs are not present in the blood of the quiescent cases and did not interfere with the peripheral cells. Horses were considered eye-healthy if the owner reported no history of eye inflammations and a routine clinical examination revealed no pathophysiological changes in the eye. Equine whole blood samples were taken from the *vena jugularis* and collected in tubes with heparin sodium (50 I.U. per ml blood; Ratiopharm, Ulm, Germany). Following the sedimentation of blood components, the leukocyte-rich plasma was used for density gradient centrifugation (room temperature (RT), 350× *g*, 25 min, brake off) with Pancoll separation solution (PanBiotech, Aidenbach, Germany). Lymphocytes were collected from the intermediate phase and washed three times with phosphate-buffered saline (PBS) (4 °C, 800× *g*, 5 min). Cells were then counted with trypan blue solution (VWR Life Science, Darmstadt, Germany) and used the same day. No experimental animals were used in this study. Collection of blood samples was permitted by the local authority (Regierung von Oberbayern, permit number: ROB-55.2-2532.Vet_03-22-37).

### 4.2. Magnetic Activated Cell Sorting (MACS) of CD4+ T Cells Using LS Columns

To obtain the CD4^+^ T cell fraction, 1 × 10^8^ lymphocytes were washed (4 °C, 800× *g*, 5 min) and resuspended in 10 mL MACS buffer (phosphate-buffered saline (pH 7.2), supplemented with 2 mM EDTA (AppliChem, Darmstadt, Germany) and 0.5% bovine serum albumin (Serva, Heidelberg, Germany)). Five µL of mouse anti-horse CD4 antibody (clone CVS4, Biorad, Feldkirchen, Germany, 1:2000) were added and incubated for 20 min at 4 °C. Ten ml of MACS buffer were added and after another washing step (4 °C, 800× *g*, 5 min), cells were resuspended in 80 µL MACS buffer per 1 × 10^7^ cells before adding 20 µL of anti-mouse IgG1 microbeads (Miltenyi Biotec, Bergisch Gladbach, Germany) per 1 × 10^7^ cells, which was incubated for 15 min at 4 °C. After that, the cells were washed again (4 °C, 300× *g*, 10 min) and resuspended in 500 µL MACS buffer to perform cell sorting. LS columns were used to perform magnetic separation (Miltenyi Biotec). Unlabeled CD4^−^ T cells were washed through the columns by adding 3 mL of MACS buffer 3 times, while magnetically labeled CD4^+^ T cells remained in the column. To collect the remaining CD4^+^ T cells, the column was removed from the magnetic field and rinsed with 5 mL of MACS buffer. The purity of the CD4^+^ T cell fraction was tested via flow cytometry after staining of 2 × 10^5^ cells of each fraction with 30 μL mouse anti-horse CD4 FITC labeled antibody (clone MCA1078F, Biorad, Feldkirchen, Germany, 1:10), which resulted in a mean purity of 98% ± 1.7% SD. (Supplementary Figure S1).

### 4.3. Flow Cytometric Analysis

CD4^+^ T cells from controls and ERU cases were stained in 96-well round-bottom plates with 4 × 10^5^ cells per well. To quantify ROS production, cells were stained with 10 µM of 2′,7′-Dichlor-dihydrofluorescein-diactetate (DCF-DA; Merck, Darmstadt, Germany). After incubation for 30 min at RT, cells were measured before and after 90 min of stimulation with PWM (Sigma-Aldrich, Darmstadt, Germany; 1 µg/mL). Measurements were performed with a NovoCyte Quanteon flow cytometer (Agilent Technologies, Waldbronn, Germany). Unstimulated cells were set to 1 and the ROS factor of stimulated cells was determined in relation accordingly. Data were analyzed using NovoExpress Software version 1.5.0 (Agilent Technologies). A representative gating strategy is shown in Supplementary Figure S2.

### 4.4. Real-Time Cell Metabolic Analysis by Seahorse XFe Analyzer

The mitochondrial metabolism of equine CD4^+^ T cells (6 controls, 5 ERU cases) was analyzed in eleven independent experiments by measuring the OCR using a Seahorse XFe Analyzer (Agilent Technologies). Dependency (Ctr, *n* = 6; ERU, *n* = 5), capacity, and flexibility (Ctr, *n* = 5; ERU, *n* = 5) were determined. One day before the assay, 24-well plates were coated with 52 µL of Poly-D-Lysine (Merck) according to the manufacturer’s protocol. A 1:1000 pre-dilution of the FCCP stock solution (carbonyl cyanide-4-phenylhydrazone; Sigma-Aldrich; 0.1 M) was prepared using a sterile XF assay buffer (Seahorse XF RPMI Medium, pH 7.4; Agilent Technologies). FCCP was then added to the cell suspension at a final concentration of 1 μM for mitochondrial uncoupling. 8 × 10^5^ cells were seeded per well in 200 µL of sterile XF assay buffer, which had been supplemented with 1 mM pyruvate, 10 mM glucose, and 2 mM L-glutamine (all three Merck). At least four wells were filled with medium to serve as background correction. Analyses were performed with three technical replicates per horse. The mean values of the technical replicates were then used for further statistical analysis. OCR was reported in units of pmol/min. After cell seeding, the plate was centrifuged at 2000 rpm for 1 min with low acceleration and low break to ensure the cells were evenly distributed across the bottom of each well. To achieve a final volume of 500 µL per well, all wells were supplemented with 300 µL of XF medium. The cell plate was incubated in a CO_2_-free incubator for 20 min before the assay. Meanwhile, the calibration plate was prepared according to the manufacturer’s instructions. Following baseline measurement, the three pathway inhibitors were sequentially injected to determine the percentage of OCR reduced by either a single inhibitor or a combination of the others. These inhibitors included 120 µM BPTES (Bis-2-(5-phenylacetamido-1,3,4-thiadiazol-2-yl)ethylsulfide), targeting the glutamine pathway, 160 µM Etomoxir for long-chain fatty acid pathway inhibition, and 80 µM UK5099, a mitochondrial pyruvate carrier inhibitor (all three from Merck). For dependency measurement, the pathway of interest was inhibited during the first injection, followed by inhibition of the two alternative pathways. For capacity, the order was reversed. Through this, the capacity and dependency were assessed for glucose, glutamine, and LCFAs using the Seahorse XF Mito Fuel Flex Test (Agilent Technologies), which allowed for the calculation of the flexibility for each fuel source. Data analysis and interpretation was carried out using the WAVE 2.6 software in accordance with the manufacturer’s manual (both Agilent Technologies).

### 4.5. Statistical Analysis

The Kolmogorov–Smirnov (KS) test was used to determine Gaussian distribution. Since analyzed data showed normal distribution (KS test was not significant; *p* > 0.05), Student’s *t*-test was used to test for statistical significance. The results were considered not significant (ns) at *p* > 0.05 and significant at *p* ≤ 0.05. Significances are indicated by asterisks with * *p* ≤ 0.05, ** *p* ≤ 0.01, and *** *p* ≤ 0.001. GraphPad Prism software (version 5.04, GraphPad Software, San Diego, CA, USA) was used for statistical analysis and graph creation.

## Data Availability

The original contributions presented in the study are included in the article/Supplementary Material, further inquiries can be directed to the corresponding author.

## Supplementary Materials

The supporting information can be downloaded at: https://www.mdpi.com/article/10.3390/ijms252111513/s1.
