# Supplementary material for: Enhanced ROS Production and Mitochondrial Metabolic Shifts in CD4+ T Cells of an Autoimmune Uveitis Model
Source: Int J Mol Sci. 2024 Oct 26;25(21):11513. doi: 10.3390/ijms252111513 (PMC11545935; doi:10.3390/ijms252111513)
Supplement: Supplementary file 1 [file ijms-25-11513-s001.zip › ijms-3250755-supplementary.pdf]

Supplementary Material

1.1 Supplementary Figures

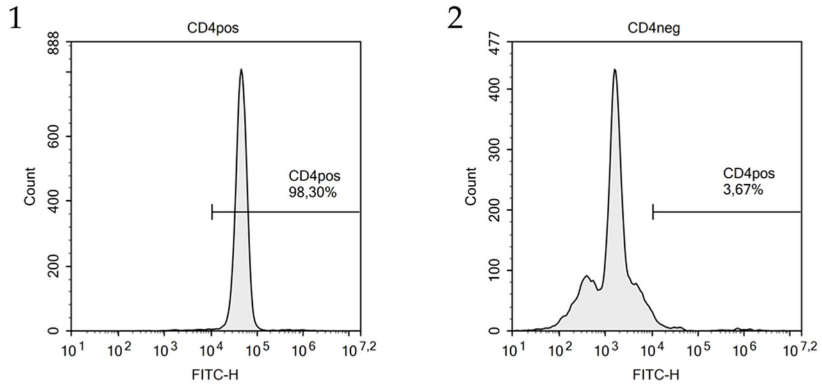

Figure S1: Purity of CD4⁺ T cell fractions after magnetic activated cell sorting (MACS). (1) Graph showing representative flow cytometry result of a CD4 positive T cell fraction, stained with mouse anti horse CD4 FITC antibody. (2) Graph showing a representative flow cytometry result of the flow-through CD4 negative T cell fraction, stained with mouse anti horse CD4 FITC antibody.

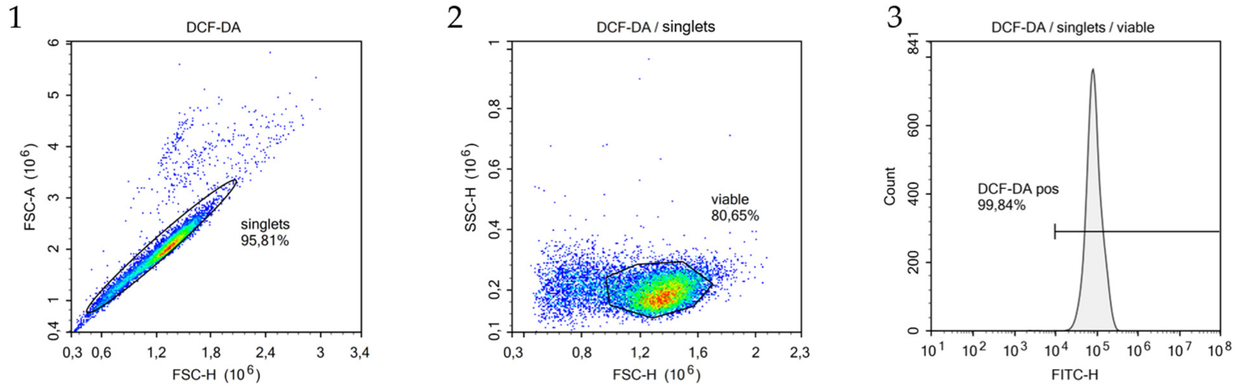

Figure S2: Representative gating strategy for flow cytometry analysis in DCF-DA staining experiments. (1) Single cells were selected in a forward scatter-height vs forward scatter-area density plot. (2) Viable cells (PI negative) were used for further analysis in (3) DCF-DA staining. Positive cells were selected by FITC-H expression.
